# Commentary: Compulsive drug use is associated with imbalance of orbitofrontal- and prelimbic-striatal circuits in punishment-resistant individuals

**DOI:** 10.3389/fncir.2020.00049

**Published:** 2020-08-25

**Authors:** Hang-Bin Zhang, Hui Zheng, Yi Zhang, Di Zhao

**Affiliations:** Shanghai Key Laboratory of Psychotic Disorders, Shanghai Mental Health Center, Shanghai Jiao Tong University School of Medicine, Shanghai, China

**Keywords:** compulsive behavior, frontostriatal networks, functional connectivity, substances use disorders, self-administration

Substance use disorders (SUDs) cause heavy economic burden and seriously challenges public security. Nearly 35 million people are receiving substance abuse treatment, and 585,000 people in the world died from overdose in 2017 (The United Nations Office on Drugs and Crime, 2019). SUDs has been widely associated with disrupted control of urges to use drugs, revealed by compulsive drug-seeking despite harmful consequences (Hyman et al., 2006; Volkow et al., 2016). Over the past two decades, researchers have shifted their focus to investigate chronic substance use induced brain plasticity at neurocircuit level underlying compulsive behaviors in addiction [e.g., cocaine (Hu et al., 2015), nicotine (Bi et al., 2017), alcohol (Grodin et al., 2018; Strosche et al., 2020), heroin (Wang et al., 2013), methamphetamine (Kohno et al., 2014)]. A recent research using resting-state functional connectivity (rsFC) has identified “go”—“stop” circuits imbalance contributing to compulsive drug use symptoms in cocaine dependents. The “go” circuits (the right ventral striatum superior-anterior prefrontal cortex/orbitofrontal cortex) are hypothesized to promote drug seeking, while the “stop” circuits (the right ventral striatum inferior–dorsal anterior cingulate cortex) are hypothesized to restrain such behaviors (Hu et al., 2015). However, a full understanding of “go”—“stop” circuits in three-stage (binge/intoxication, withdrawal/negative affect, and preoccupation/anticipation) addiction cycle is still poorly understood. Furthermore, neurocircuit mechanisms that account for the heterogeneity in drug response in the preclinical study still remain unknown.

These understandings were addressed in a recent study published in the journal of Proceedings of the National Academy of Sciences of the United States of America by Hu et al. (2019). The research employed a longitudinal functional magnetic resonance imaging (fMRI) design to dissect neurocircuit mechanisms underlying addiction in the self-administration (SA) rat model. The animal experimental procedure paralleled the stages of compulsive drug taking cycle in methamphetamine dependents, including SA phase as binge/intoxication stage and foot-shock punishment as negative affect/withdrawal stage. All rats were scanned by fMRI at baseline and after these key time points above. Seed based rsFC analyses were applied later. In consideration of the individual difference on compulsive behavior, rats were differentiated to shock-resistant (SR) and shock-sensitivity (SS) subgroup rats by “compulsivity index” (CI, a ratio of meth infusion on last foot shock day versus last SA day). In SA behavior, no difference was found between two subgroup rats before and after stable SA. During punishment phase, the shock-resistant (SR) subgroup rats showed continuous lever pressing with higher CI, while the shock-sensitivity (SS) subgroup rats controlled drug use with lower CI. In neurocircuitry level, the rsFC of orbitofrontal-medial striatum (OFC-MS) “go” circuit increased and prelimbic-ventral striatum (PrL-VS) “stop” circuit decreased until the end of SA phase in both two subgroups. While in foot-shock punishment stage, only the rats of SR group showed the rsFC of go circuit decreased and stop circuit increased, which consisted with their SA behavior. Briefly, the balance between go and stop circuit (indicated by go—stop) differed in two subgroups, and significantly associated with compulsive level. These results showed that when facing adverse consequences (e.g., punishment), addictive individuals diversified the compulsive behavior and go-stop circuit.

## Limitations and Future Directions

Drug addiction is characterized by disruption in finely balanced brain networks. Over the two decades, the animal models (Shippenberg and Koob, 2002) and human neuroimaging studies (Hu et al., 2015) has facilitated the understanding of neurobiology of addiction. In a recent review, Koob and Volkow have proposed that three domains (motivational “wanting”-incentive salience, emotional “liking”-negative emotional state, and executive control) mediated by three neurocircuits (basal ganglia, extended amygdala, and prefrontal cortex) (Koob and Volkow, 2016). A long stand of human studies had indicated the orbitofrontal cortex (the hub of go circuit) was essential for regulating incentive salience when a drug cue was presented to the drug dependents (Volkow and Fowler, 2000; Clarke et al., 2008; Hart et al., 2014). And evidence from rodent research has demonstrated that drug-related reinstatement involved by stop circuit that links PrL to VS, which correspond with anterior cingulate cortex (ACC) in human (McFarland and Kalivas, 2001). Hu and colleagues have identified the balance of “go-stop” circuit to produce compulsive-like habits by combining the merits of animal model and brain imaging in individuals. However, in the study, animal model could not fully emulate the addiction cycle (e.g., the preoccupation/anticipation stage, which is a pivotal stage of relapse in humans) and complex emotional dysregulation correlated with withdrawal and protracted abstinence (de Wit, 2009; McEwen and Morrison, 2013; Maier et al., 2015) in human. There is also evidence indicating withdrawal from drug can cause fatigue, irritability, insomnia, and psychological symptoms (e.g., depression and anxiety; Koob and Le Moal, 1997). Future study to ascertain the shared and distinct neurocircuit mechanisms between depression/anxiety and addiction would be of interest.

In conclusion, this research has suggested that the imbalance of brain circuits plays an important role in maladaptive behavior in drug dependents (Everitt and Robbins, 2016). These findings have underscored that the balance of resting-state functional connectivity would be a potential neuroimaging biomarker to forecast future addiction relapse, and provide neurocircuit candidate for exploring the genetic basis of endophenotypes related to addiction. Moreover, these results extended previous understanding of fundamental brain circuits involved in addiction cycle and opened for a principled way to develop individualized treatment in SUDs. Prior works from our lab have focused on stimulating the dorsolateral prefrontal cortex (DLPFC), a hub of frontal-striatal circuitry that is dysfunctional in SUD individuals (Liu et al., 2017, 2019; Shen et al., 2017; Zhao et al., 2020). Our results have manifested through either activation of left DLPFC or inhibition of the right DLPFC may reduce craving and alleviate symptoms of depression in methamphetamine dependents (Zhao et al., 2020). Another strand studies from Hanlon lab have suggested that stimulating left frontal areas can induce selective changes in ventromedial prefrontal cortex (VMPFC), the striatum, and ACC thus change drug cue reactivity in cocaine dependent- and alcohol dependent patients (Hanlon et al., 2015, 2016, 2017, 2018). These empirical results showed that it would be possible to attenuate stop circuit through targeting frontal areas or ACC, which may lay a foundation for future clinical trials for treating SUDs.

## Author Contributions

H-BZ, HZ, YZ, and DZ conceived the idea and wrote the manuscript together. All authors listed have made a substantial, direct and intellectual contribution to the work, and approved it for publication.

## Conflict of Interest

The authors declare that the research was conducted in the absence of any commercial or financial relationships that could be construed as a potential conflict of interest.
